# Long-term follow-up of an IgA nephropathy cohort: outcomes and risk factors

**DOI:** 10.1080/0886022X.2022.2152694

**Published:** 2023-01-23

**Authors:** Liliana Gadola, María Jimena Cabrera, Mariela Garau, Ruben Coitiño, María Haydée Aunchayna, Oscar Noboa, María Asunción Alvarez, Sylvia Balardini, Graciela Desiderio, Nelson Dibello, Alejandro Ferreiro, Soledad Giró, Leonella Luzardo, Alfredo Maino, Lucía Orihuela, María Gabriela Ottati, Andrés Urrestarazú

**Affiliations:** a Programa de Prevención y Tratamiento de las Glomerulopatías (PPTG), Montevideo, Uruguay; b Centro de Nefrología. Hospital de Clínicas, Facultad de Medicina, Universidad de la República, Montevideo, Uruguay

**Keywords:** IgA nephropathy, risk factors, kidney replacement therapy, outcomes

## Abstract

**Aim:**

IgA nephropathy (IgAN), the most common glomerulopathy worldwide and in Uruguay, raised treatment controversies. The study aimed to analyze long-term IgAN outcomes and treatment.

**Methods:**

A retrospective analysis of a Uruguayan IgAN cohort, enrolled between 1985 and 2009 and followed up until 2020, was performed. The Ethics Committee approved the study. The inclusion criteria were (a) biopsy-proven IgAN; (b) age ≥12 years; and (c) available clinical, histologic, and treatment data. The patients were divided into two groups, with immunosuppressive (IS) or without (NoIS) treatment. Outcomes (end-stage kidney disease/kidney replacement therapy [ESKD/KRT] or all-cause death) were obtained from mandatory national registries.

**Results:**

The study population included 241 patients (64.7% men), median age 32 (19.5) years, baseline blood pressure <130/80 mmHg in 37%, and microhematuria in 67.5% of patients. Baseline proteinuria, glomerulosclerosis, and a higher crescent percentage were significantly more frequent in the IS group. Proteinuria improved in both groups. Renal survival at 20 years was 74.6% without difference between groups. In the overall population and in the NoIS group, bivariate Cox regression analysis showed that baseline proteinuria, endocapillary hypercellularity, tubule interstitial damage, and crescents were associated with a higher risk of ESKD/KRT or death, but in the IS group, proteinuria and endocapillary hypercellularity were not. In the multivariate Cox analysis, proteinuria in the NoIS group, crescents in the IS group and tubule interstitial damage in both groups were independent risk factors.

**Conclusion:**

The IS group had more severe risk factors than the NoIS group but attained a similar outcome.

## Introduction

IgA nephropathy (IgAN), the most common biopsied primary glomerulopathy worldwide [1], particularly in Asia, has also been the most or second-most common biopsied glomerulopathy in Uruguay (3–12 per million population [pmp]) [2,3]. The incidence differences may be explained by genetic and environmental causes and different policies on mass urine screening, timely referral to a nephrologist for asymptomatic urinary abnormalities, and kidney biopsy (KB) criteria [1,4]. Clinical presentation is variable, from asymptomatic urinary abnormalities to rapidly progressive glomerulonephritis, as well as a slowly progressive clinical course of deteriorating kidney function [4], likely because there are different pathogenic mechanisms. Therefore, the individual prognosis is difficult to assess, and prognostic online risk calculators have been developed [5–9] but are not recommended for therapy decisions. Treatment is under discussion and initiating supportive therapy is widely accepted [10], with renin-angiotensin-aldosterone system blockers (RASBs) as a cornerstone [11,12], but the additive benefit of immunosuppressive (IS) agents remains controversial [10–16]. Additionally, histology is not considered in the choice of immunosuppression; only a crescent percentage >50% was included as a treatment indication in the recent KDIGO Guidelines [10], but its inclusion was controversial [17,18].

The Uruguayan Registry of Glomerulopathies (URG), started in 1970, includes data from all kidney-biopsied patients in the country, with an average biopsy rate of 58 pmp/year [3]. Glomerular disease reports have been mandatory since 2000. URG is maintained by the ‘Programa de Prevención y Tratamiento de las Glomerulopatías’ (Glomerular Disease Prevention and Treatment Program) (PPTG), which also establishes national guidelines that are periodically updated [19], taking into account the national epidemiology and international guidelines from KDIGO [10]. This national registry, which currently includes over 5000 cases, offers an opportunity for long-term studies on glomerulopathy outcomes. Historically, national guidelines on IgAN [19] have always suggested the use of histologic lesions for therapeutic decisions, which is controversial, so it would be worth analyzing outcomes.

The aim of the study was to analyze the long-term outcomes and IS treatments in an IgAN cohort.

## Materials and methods

A retrospective analysis of an IgAN cohort (over 12 years old) included in the URG between 1 January 1985 and 31 December 2009, and followed up until 31 December 2020, was performed.

The URG obtains data from all pathologists who perform native KBs in the country and registers clinical data provided by attending nephrologists at the time of KB. It is included in the National Renal Healthcare Program of Uruguay (NRHP-UY). The URG resides in a Data Center of the Nephrology Department, Universidad de la República, and is confidentially cross-referenced with the mandatory National Registry of end-stage kidney disease/kidney replacement therapy (ESKD/KRT) and with the National Death Registry (all-cause mortality). Kidney replacement therapy (hemodialysis, peritoneal dialysis, and kidney transplant) has been freely available throughout the country since 1979. Patients were deemed alive on 31 December 2020, if they were not admitted to KRT, or did not die, before that date (as per the mentioned mandatory national registries).

Age, sex, systolic and diastolic blood pressure (SBP/DBP), proteinuria, serum creatinine, and estimated glomerular filtration rate (eGFR) calculated by the CKD-EPI formula [20] at inclusion were registered. Data were included as reported to URG by treating nephrologists. The eGFR was classified into three groups: >60 mL/min/1.73 m^2^, 30–60 mL/min/1.73 m^2^, and <30 mL/min/1.73 m^2^.

Proteinuria data on evolution (not always at the end of follow-up) were available in 177 patients. Proteinuria data were classified into three groups: mild (<0.75 g/L or 1 g/d), moderate (0.75–3 g/L or 1–3.5 g/d), and severe (>3 g/L or 3.5 g/d) (as a nationwide retrospective study, available data were heterogeneous).

The following KB data were also registered: glomeruli number, mesangial hypercellularity, endocapillary hypercellularity, glomerulosclerosis, tubular atrophy/interstitial fibrosis, and crescent percentage (retrospectively classified according to MEST-C [21]). Crescent percentages were classified into four groups: none, 1–10%, 11–29%, and ≥30%.

RASBs, either angiotensin-converting enzyme inhibitors (ACEIs) or angiotensin receptor blockers (ARBs), and IS (corticosteroids, azathioprine, cyclophosphamide, or others) treatments were registered. Data on dose, time of initiation, and treatment duration were not available.

**Figure 1.**
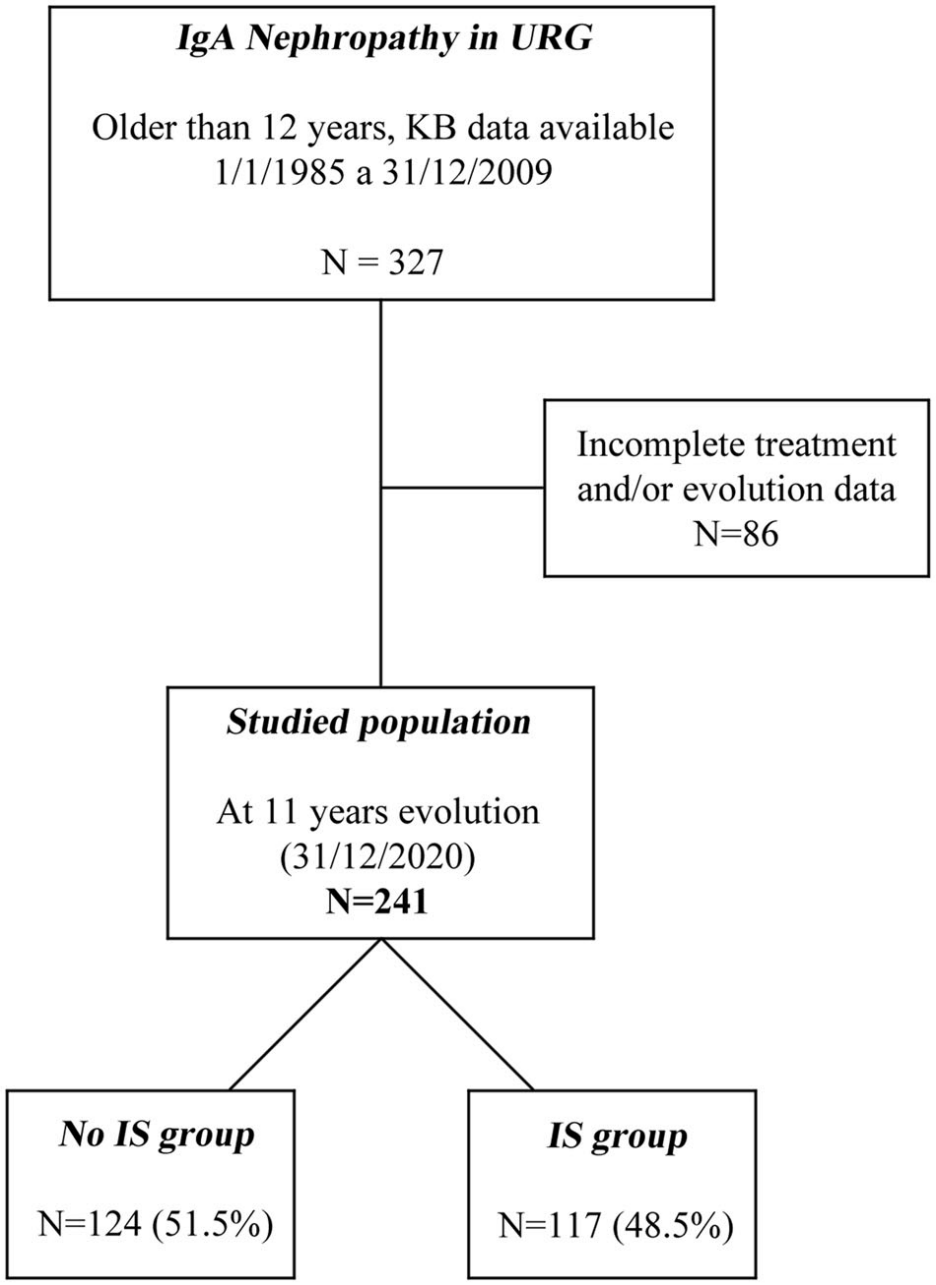
Algorithm with the studied population, immunosuppressive (IS), and no immunosuppressive (NoIS) groups.

The cohort inclusion criteria were (a) biopsy-proven IgAN by light microscopy and immunofluorescence, observed by experienced nephropathologists; (b) 12 years of age or older at KB; and (c) available data on clinical variables and KB findings at inclusion and the treatment received (Figure 1).

The baseline data of the studied population and RASB treatment are detailed in Table 1.

**Table 1.** Baseline data.

	Global population (*n* = 241)			
	All (241)	No IS (124)	IS (117)	*p* Value
Age (years) median (pc 25–75)	32.0 (21.5–40.9)	31.7 (20.0–41.8)	32.0 (23.0–40.0)	*0.762
Sex (M) (n, %) *n* = 241	156 (64.7%)	73 (58.9%)	83 (70.9%)	**0.05
SBP (mmHg) median (pc 25–75) data = 232	130 (120–150)	130 (110–142)	130 (120–50)	*0.084
DBP (mmHg) median (pc 25–75) data = 232	80 (70–90)	80 (70–90)	80 (70–955)	*0.205
BP < 130/80 mmHg (*n*, %) data = 232	85 (37.0%)	49 (42.2%)	36 (31.6%)	**0.094
Urine protein Groups (*n*, %)				**0.002
<0.75 g/L or 1 g/d	90 (38.1%)	57 (47.5%)	33 (28.4%)	
≥0.75 g/L or 1 g/d and ≤3 g/l or 3.5 g/d	106 (44.9%)	51 (42.5%)	55 (47.4%)	
>3 g/L or 3.5 g/d	40 (16.9%)	12 (10%)	28 (24.1%)	
Hematuria Groups (*n*, %) data = 231				**0.852
No	29 (12.6%)	15 (12.6%)	14 (12.5%)	
Microhematuria	156 (67.5%)	62 (68.9%)	74 (66.1%)	
Macrohematuria	46 (19.9%)	22 (18.5%)	24 (21.4%)	
eGFR (mL/min/1.73 m^2^) median ( pc 25–75) data = 232	77.1 (53.9-99.7)	79.3 (62.7-101.4)	73.2 (44.3-96.4)	*0.053
eGFR Groups (*n, %*) data = 232				**0.081
>60 mL/min/1.73 m^2^	164 (71.0%)	90 (77.6%)	74 (71.0%)	
30–60 mL/min/1.73 m^2^	53 (22.9%)	20 (17.2%)	33 (28.7%)	
<30 mL/min/1.73 m^2^	14 (6.1%)	6 (5.2%)	8 (7.0%)	
Histologic data (*n*, %)				
Endocapillary hypercellularity (yes) data =222	60 (27%)	27 (24.8%)	33 (29.2%)	**0.450
Glomerulosclerosis (Yes) data =62	29 (46.8%)	9 (31%)	20 (60.6%)	**0.020
Tubulo-interstitial damage groups				**0.206
<25%	161 (73.9%)	84 (79.2%)	77 (68.6%)	
25–50%	50 (22.9%)	19 (17,9%)	31 (27.7%)	
>50%	7 (3.2%)	3 (2.8%)	4 (3.6%)	
Crescents groups (*n*, %)				**0.000
None	119 (53.6%)	83 (76.1%)	36 (31.0%)	
1–10%	44 (19.8%)	13 (11.9%)	31 (27.4%)	
11–29%	37 (16.7%)	8 (7.3%)	29 (25.7%)	
≥30%	22 (9.9%)	5 (4.6%)	17 (15.0%)	
RASBs (yes) (*n*, %)	182 (85.4%)	85 (80.2%)	97 (90.7%)	**0.03

SBP: systolic blood pressure; DBP: diastolic blood pressure; BP: blood pressure; M: male; pc: percentile; RASBs: renin-angiotensin-aldosterone system blockers.

*Chi^2^ test, **Mann–Whitney test *p* value.

The patients included were divided into two groups based on the IS treatment received: (a) the no IS group (NoIS), patients who did not receive corticosteroids or other IS therapies (*n* = 124, 51,5%), and (b) the IS group, patients who received corticosteroids and/or other IS therapies (*n* = 117, 48.5%).

All IS treatments were combined into one group because all patients in the IS group received corticosteroids (prednisone and/or methylprednisolone), but only 33 patients (28.7%) received them exclusively. Corticosteroids were combined with cyclophosphamide (*n* = 24, 20.9%), azathioprine (*n* = 70, 60.9%), or mofetil mycophenolate (*n* = 9, 7.8%). Some patients received cyclophosphamide and azathioprine.

The primary outcomes analyzed were the initiation of chronic maintenance dialysis or kidney transplant (ESKD/KRT) or all-cause death. The secondary outcome was proteinuria change in evolution.

### Statistical analysis

For the descriptive analysis, data are presented as summary measures (median and interquartile range (IQR), percentage, and 95% confidence interval [CI]). Tests adjusted to variable nature and distribution were used for the statistical inference analysis (Mann–Whitney, chi-square). Survival was estimated using the Kaplan–Meier method and compared across groups using the log-rank test. Risk estimation was conducted by calculating the hazard ratio (HR) by multivariate Cox regression, with the corresponding 95% CI, adjusted for variables that were not interassociated (Cramer’s *V*  <0.25) and were significantly associated with the combined event in the bivariate Cox regression analysis.

In every case, the null hypothesis was rejected at *p* < 0.05 or nonoverlapping 95% CIs. IBM SPSS version 15.0 software[Q] was used for the analysis.

### Ethics

The Hospital de Clínicas, Universidad de la República, Ethics Committee approved the study protocol. Strict confidentiality was assured in all cases. Glomerular disease notification to the URG is mandatory by a Ministry of Health ordinance (324/2000). Informed consent was obtained for epidemiological research at the moment of KB or when patients were included in the National Renal Healthcare Program of Uruguay (NRHP-UY). The Ethics Committee authorized the review of clinical data of those patients who died without signing informed consent.

## Results

There were 327 patients older than 12 years included in the URG between 1 January 1985, and 31 December 2009, followed up until 31 December 2020, with histologically confirmed IgAN (10.8%). Because 86 had no treatment registry or evolution data, they were excluded, so 241 patients were finally included in this study (studied population) (Figure 1).

Baseline clinical and histologic data are included in Table 1.

The median age (pc 25–75) of the population was 32 (21.5–40.9) years, there were 156 males (64.7%), and the baseline SBP (median, pc 25–75) was 130 (120–150) mmHg and DBP was 80 (60–90) mmHg. Only 37% of patients had blood pressure <130/80 mmHg at presentation, without differences between IS groups (NoIS and IS). Microscopic hematuria was observed in 67.5% of patients and macroscopic hematuria in 19.9% of patients, without differences between groups. Moderate and severe proteinuria, glomerulosclerosis and a higher percentage of crescents were significantly more frequent in the IS group (Table 1).

### Outcome

**Figure 2.**
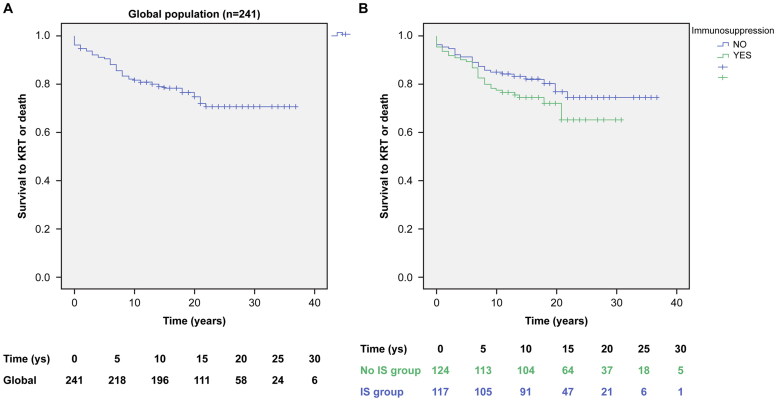
Studied population renal survival curves. A. Kaplan–Meier renal survival curve (Event = ESKD/KRT or death). (B) Kaplan–Meier renal survival curve (Event = ESKD/KRT or death) according to immunosuppressive treatment, and the number of exposed patients at each time. (Log-rank *p* = 0.127). Mean survival time to combined event (CI 95%) was 28.9 (27.1–30.8) years the in global population, 30.2 (27.9–32.6) years in the No IS group, and 23.6 (21.4–25.8) years in the IS group.

Survival to ESKD/KRT or death (combined event) of the studied population was 90.9% at 5 years (three deaths), 81.7% at 10 years (six deaths), 78.6% at 15 years (nine deaths), and 74.6% at 20 years (ten deaths), without differences between the NoIS and IS groups (log-rank, *p* = 0.127) (Figure 2). The mean survival time to combined event (CI 95%) was 28.9 (27.1–30.8) years in the global studied population, 30.2 (27.9–32.6) years in the NoIS group, and 23.6 (21.4–25.8) years in the IS group.

**Figure 3.**
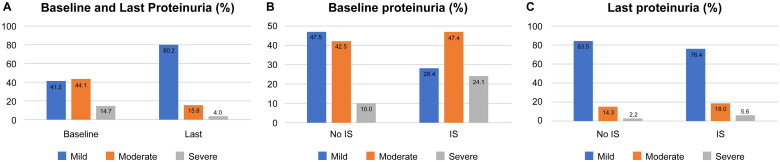
(A) Baseline and Last available proteinuria data (*n* = 177). Levels: Mild <0.75 g/L o 1 g/d, Moderate between 0.75 g/L or 1 g/d and 3 g/L or 3.5 g/d and Severe > 3 g/L or 3.5 g/d. (B) Baseline and (C). Last proteinuria, in No Immunosuppressive (No IS, *n* = 88) and Immunosuppressive (IS, *n* = 89) Groups. Baseline *vs* Last proteinuria was significantly different in the No IS group (Chi^2^ *p* = 0.04) and in the IS group (Chi^2^ *p* = 0.007). The time between baseline and last data (median, IQR): Studied population 65 (82) months, IS group 64 (74) months, no IS group 65 (93) months.

In the patients with available proteinuria data on evolution (not at the end of follow-up) (*n* = 177), mild proteinuria was significantly more frequent in the last available data *vs.* baseline data in both the NoIS and IS groups (Figure 3). In the IS group, baseline proteinuria was more frequently moderate or severe (71.5%) *vs.* the NoIS group (52.5%) (chi-square, *p* = 0.002), but proteinuria significantly diminished in evolution in both groups. The last available data showed moderate or severe proteinuria in 16.5% of the NoIS group (chi-square, *p* = 0.04 *vs.* baseline) and 23.6% of the IS group (chi-square, *p* = 0.007 *vs.* baseline) (Figure 3). There was no significant difference between the last available proteinuria data in the NoIS group *vs.* the IS group (chi-square, *p* = 0.364). The time between baseline and last available data (median, IQR) was 65 (82) months in the entire population, 64 (74) months in the IS group, and 65 (93) months in the NoIS group (Figure 3(A–C).

RASBs (either ACEIs or ARBs) were administered to 182 patients (85.4%) and 117 patients (48.5%) received IS therapy. In the IS group, all patients received glucocorticoids (prednisone and/or methylprednisolone), 33 patients exclusively (28.7%), and the rest received cyclophosphamide (*n* = 24, 20.9%), and/or azathioprine (*n* = 70, 60.9%), or mofetil mycophenolate (*n* = 9, 7.8%).

In the studied population, a higher risk of the combined event (ESKD/KRT or death) was associated (Cox bivariate regression analysis) with higher baseline proteinuria, eGFR <60 mL/min/1.73 m^2^, endocapillary hypercellularity, tubule interstitial damage ≥25%, and crescents in ≥30% of glomeruli (Table 2A). The same risk factors were observed in the NoIS group, but in those who had received IS treatment (IS group), neither proteinuria nor endocapillary hypercellularity was associated with a higher risk of the combined event (Table 2A).

**Table 2A.** Risk to ESKD/KRT or death in the Global studied population, in the NoIS and IS groups. Bivariate Cox regression analysis.

	All (241)			No IS group (124)			IS group (117)		
	HR	CI 95%	*p*	HR	CI 95%	*p*	HR	CI 95%	*p*
Age (years) (continuous)	1.018	0.999–1.037	0.068	1.017	0.989–1.046	0.229	1.018	0.991–1.045	0.190
Sex (Ref. male)	1.156	0.678–1.971	0.594	0.515	0.215–1.233	0.136	2.460	1.227–4.933	0.011
BP initial (Ref < 130/80 mmHg)	1.365	0.770–2.418	0.287	1.671	0.721–3.873	0.231	1.032	0.471–2.262	0.937
eGFR initial (Ref > 60 mL/min/1.73 m^2^)			0.000			0.000			0.000
30–60 mL/min/1.73 m^2^	2.898	1.584–5.301	0.001	2.189	0.839–5.713	0.109	3.405	1.491–7.775	0.004
<30 mL/min/1.73 m^2^	20.123	9.831–41.188	0.000	15.069	5.254–43.223	0.000	24.508	8.798–68.267	0.000
Proteinuria initial groups (Ref. mild)			0.006			0.000			0.559
Moderate	2.244	1.144–4.399	0.019	2.826	0.995–8.025	0.051	1.575	0.653–3.798	0.312
Severe	3.375	1.578–7.218	0.002	13.463	4.300–42.149	0.000	1.172	0.410–3.349	0.767
Endocapillary hypercellularity (Ref. NO)	2.085	1.207–3.604	0.008	2.277	1.009–5.138	0.048	1.899	0.906–3.980	0.089
Glomerulosclerosis (Ref. NO)	1.955	0.639–5.980	0.240	0.747	0.078–7.182	0.800	2.512	0.523–12.154	0.249
Tubule-interstitial damage groups (Ref. <25%)			0.000			0.000			0.000
25–50%	4.403	2.463–7.873	0.000	3.286	1.359–7.941	0.008	5.243	2.312–11.887	0.000
>50%	26.115	10.617–64.236	0.000	16.027	4.353–59.001	0.000	41.067	10.816–155.929	0.000
Crescents group (Ref. none)			0.000			0.048			0.000
≤10%	0.834	0.336–2.070	0.695	2.008	0.665–6.067	0.217	0.389	0.078–1.933	0.248
11 a 29%	1.957	0.961–3.985	0.064	1.388	0.317–6.081	0.663	2.114	0.767–5.823	0.148
≥30%	6.664	3.329–13.342	0.000	5.680	1.623–19.876	0.007	6.697	2.414–18.582	0.000

BP: blood pressure; eGFR: estimated glomerular filtration rate; Ref: reference

In the multivariate Cox regression analysis, adjusted as explained in Methods, the independent risk factors in the entire population and in the NoIS group were proteinuria (HR 2.254, CI 95% 1.001–5.076 and HR 13.724, CI 95% 3.835–49.118, respectively) and tubule interstitial damage ≥25% (HR 3.529, CI 95% 1.869–6.661 and HR 3.077, CI 95% 1.162–8.147, respectively), but in the IS group, tubule interstitial damage ≥25% (HR 3.089, CI 95% 1.273–7.498) and crescent percentage ≥30% (HR 4.233, CI 95% 1.461–12.269) were associated with a higher risk of the combined event (Table 2B).

**Table 2B.** Risk to ESKD/KRT or death in the studied population (all), no immunosuppressive (NoIS) and immunosuppressive (IS) groups by multivariate Cox regression analysis.

	All (241)*			No IS group (124)*			IS group (117)**		
	HR	CI 95%	*p*	HR	CI 95%	*p*	HR	CI 95%	*p*
Proteinuria initial groups (Ref. mild)			0.145			0.000			
Moderate	1.580	0.757–3.299	0.223	2.211	0.747–6.542	0.152			
Severe	2.254	1.001–5.076	0.050	13.724	3.835–49.118	0.000			
Endocapillary hypercellularity (Ref. NO)	1.394	0.750–2.591	0.293	1.652	0.585–4.665	0.343			
Tubule-interstitial damage groups (Ref. <25%)			0.000			0.048			0.000
25–50%	3.529	1.869–6.661	0.000	3.077	1.162–8.147	0.024	3.089	1.273–7.498	0.012
>50%	13.429	4.971–36.280	0.000	3.326	0.626–17.681	0.159	28.559	7.218–112.999	0.000
Crescents group (Ref. none)			0.223			0.193			0.013
≤10%	0.878	0.345–2.234	0.785	2.874	0.792–10.424	0.108	0.522	0.103–2.650	0.590
11 a 29%	1.331	0.630–2.811	0.454	0.912	0.193–4.302	0.907	1.849	0.669–5.108	0.232
≥30%	2.185	0.984–4.848	0.055	4.086	0.836–19.977	0.082	4.233	1.461–12.269	0.005

*Adjusted to proteinuria, endocapillary hypercellularity, tubule-interstitial damage, and crescents percentage.

**Adjusted to sex, tubule-interstitial damage and crescents percentage as proteinuria and endocapillary hypercellularity were not significant in bivariate Cox analysis and the glomerular filtration rate was associated with tubule-interstitial damage (Cramer’s *V* = 0.438).

ESKD/KRT: end-tage kidney disease/kidney replacement therapy; HR: Hazard ratio; CI: confidence interval: Ref: reference

Patients in the groups with higher crescent percentages received more frequent IS treatment (Table 3A) and had poorer survival (log-rank *p* = 0.000) (Figure 3). In the group with 1–10% crescents (*n* = 44), 31 patients received IS and showed a significantly lower risk (HR 0.179, CI 95% 0.033–0.980) than those in the NoIS group (Cox bivariate regression analysis, *p* value = 0.047) (Table 3B), but it was not significantly associated in the multivariate Cox regression analysis (Supplemental Table 1).

## Discussion

An Uruguayan biopsy-proven IgAN cohort, started in 1985, with an extended follow-up (at least 11 years) was studied. To the best of our knowledge, this is the oldest and largest Latin American IgA cohort outcome report. The survival (to ESKD/KRT or death) was 81.7% at 10 years and 74.6% at 20 years, similar to that reported in other cohorts [4–8].

There are scarce data from Latin America [22–28] and the IgAN frequency reported varied between 6% in a Northern Brazilian cohort and 20.9% of KBs in Colombia, with wide differences across regions and over time [22–29]. O’Shaughnessy et al. [29], in an international survey of biopsied glomerulopathies (that included 3 Latin American cohorts from Mexico, Brazil, and Colombia), observed that IgAN was not the most common glomerulopathy diagnosed in Latin America (only 6.1%) and had a male predominance. It was the most frequent glomerulopathy in Europe (22.1%) and Asia (39.5%). They concluded that there were differences between geographical regions, probably as a consequence of environmental factors and local biopsy policies, not only because of race/ethnicity [29]. In Uruguay, the IgAN frequency has increased progressively [3]. It was 10.8% of biopsy-proven glomerulopathies in this studied cohort time frame. This may be because the Uruguayan population is 88% Caucasian [30], persistent asymptomatic urinary abnormalities are considered an indication for KB [2,3] and there is a mandatory registry of glomerulopathies (URG) [3].

This IgAN cohort’s clinical presentation is similar to that reported internationally [1,4–8]; the patients were young (median age, 32 years) and predominantly men (64.7%) with proteinuria, hematuria, and hypertension (Table 1). Crescents were frequently observed in this cohort (46.4%) (Table 1), while they were observed in only 11% of the VALIGA cohort [5]. According to national guidelines, crescents were considered an indication for IS treatment (steroids and cyclophosphamide followed by azathioprine from 6 to 18 months on tapering doses) in this study time frame [19]. This therapeutic choice is currently under debate [10,15].

RASBs were frequently administered (75.5%) (Table 1), but this rate was slightly under that reported by other retrospective cohorts [5,9,31–33], probably because this cohort started in 1985 and, as an example, VALIGA (which reported 86% RASB treatment) [5] started in 2004.

### Outcome

The survival to the combined event (ESKD/KRT or death) was 81.7% at ten years and 74.6% at 20 years, similar to that reported by other authors [4–9,31–33] in this nephropathy with a long and variable evolution. International studies [13] reported an ESKD incidence of 10–60% at 10 years and 40% at 20 years. Moriyama et al. [31,32] found a renal survival of 84.3%–87.5% at 10 years and 66.6–72.6% at 20 years. IgAN has geographic variations [1,4,29] and as far as we know, there are no long-term Latin American IgAN outcome reports.

### Risk factors for ESKD/KRT or death

According to IgAN clinical presentation and histologic damage [10], many risk factors have been described, and several predictive tools have been developed [5–9]. In this study, the risk factors for the combined event of ESKD/KRT or death, according to the bivariate Cox regression analysis, were eGFR < 60 mL/min/1.73 m^2^, severe nephrotic-range proteinuria, endocapillary hypercellularity, tubule interstitial damage higher than 25% and crescents in 30% or more of glomeruli (Table 2A). As a retrospective study, started in 1985, mesangial proliferation was not reported according to the Oxford classification, so it was not registered, and there were glomerulosclerosis data of only 62 cases, so the MEST-C score [21] could not be calculated. These risk factors were similar to those observed in other cohorts [25,26]. Oxford’s classification [8,21] demonstrated the importance of histologic damage in prognosis evaluation. An online application, recently developed, estimates renal function outcome [6,7] according to clinical and histologic (MEST-C) baseline data (https://qxmd.com/calculate/calculator_499/international-igan-prediction-tool). Despite these findings, there is no consensus on considering histologic data when choosing IgA therapy in an individual case [10].

Baseline eGFR was strongly associated with tubule interstitial damage (Cramer’s *V* = 0.438) and with ESKD/KRT and death in the bivariate Cox regression analysis in all groups (Table 2A). In a previous study of this cohort, serum creatinine ≥2.5 mg/dL was associated with a poor outcome [34], similar to that reported by others [35], so it would likely be a point of no return.

As many authors have observed, the degree and persistence of proteinuria are associated with renal function outcome and are the main risk factors for progressive chronic kidney disease and ESKD/KRT [36–38]. Usually, both aspects were included in the area under the curve of proteinuria on the follow-up time [37–40]. As this was an observational study over a long period, these data were unavailable; proteinuria was expressed in different forms, so it was reclassified as previously described in the Methods. With these limitations, severe baseline proteinuria was significantly associated with a poor outcome (ESKD/KRT or death) (HR 3.375 CI 95% 1.578–7.218 in the entire cohort, and HR 13.463 CI 95% 4.300–42.149 in the NoIS group) except in the IS group (Table 2A). The degree of proteinuria diminished significantly between the baseline and the last available data (Figure 3(A)) in both groups with or without immunosuppression (Figure 3(B,C)). The last reported proteinuria was mostly mild, under 1 g/day or 0.75 g/L. As several authors described, proteinuria improvement may be associated with better renal function outcomes [37–39] by different pathogenic mechanisms and be considered a surrogate endpoint in IgAN [39,40]. This fact may partially explain why the degree of proteinuria was not significantly associated with the risk of the combined event in the IS group (Table 2A), as the attending nephrologists may prescribe IS and optimize treatment in both groups to achieve a similar outcome.

### Histologic lesions

More severe histologic lesions were observed in the IS group (Table 1) and their association with outcomes (as risk factors) was analyzed (Table 2A and B) as previously mentioned. Glomerulosclerosis data were scarce, so it was not included in the Cox regression risk analysis although it is a recognized, irreversible risk factor.

**Figure 4.**
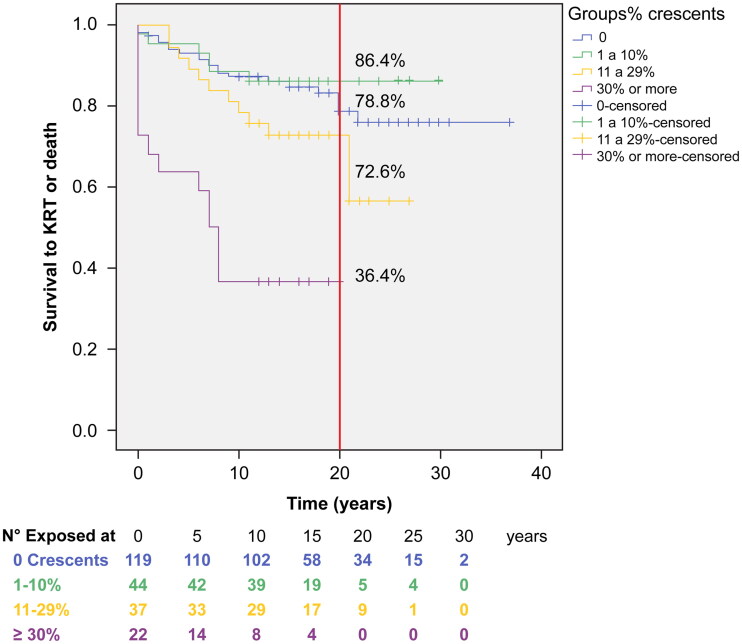
Survival to KRT or death according to crescent’s percentage in KB. Global Mean survival (CI 95%) was 28.7 (26.7–30.7) years, No-crescents group was 30.8 (28.4–33.2) years and for the ≥30% crescents was 9.4 (5.9–12.9) years. Survival at 20 years was 78.8% (group without crescents), 86.4% (group with 1–10% crescents), 72.6% (group with 11–29% crescents), and 36.4% (group ≥30% crescents). Log-rank *p* = 0.000.

Crescents were frequently observed (46.4%) in this cohort, even in slightly higher percentages than usually reported in other international cohorts [6,17,41–46], which varied from 11% in VALIGA [5] to 44.3% [41]. Nevertheless, if 30% or more of glomeruli had crescents, the risk of the combined event was significantly higher (Table 2A). The presence of crescents was also associated with poor renal function outcomes in many studies [41–43]. In the present cohort, in groups with increasing crescent percentages, the survival time to ESKD/KRT or death decreased (Kaplan–Meier curves, log-rank *p* < 0.001, Figure 4), as was observed by other authors [17,41], at percentages as low as 5% [46]. According to KDIGO Guidelines, crescents are considered an indication for IS treatment only if they are observed in more than 50% of the glomeruli (a ‘crescentic form’ treated as a ‘rapidly progressive form’) [10]. However, this decision was controversial, as several studies included groups with less than 50% crescents that received IS treatment, mainly steroids, with better outcomes [47,48]. In the present cohort, the IS group included more patients with crescents (Table 1), and the higher the crescent percentage was, the more frequently IS treatment was prescribed (Table 3) as a consequence of the adherence to historic national guidelines that take into account histologic lesions (as extracapillary proliferation) to make treatment decisions [19]. The outcome observed (74.6% survival free of ESKD/KRT or death at 20 years) may support this therapeutic choice, as other authors considered [13,17,46], even though survival was not different in the IS and NoIS groups, as was previously discussed.

**Table 3.** Immunosuppressive treatment (IS) and crescents percentage group. A Treatment according to crescents percentage groups. (B) Risk to ESKD/KRT or death. Bivariate Cox regression analysis according to IS (reference NoIS).

	0	1–10%	11–29%	≥30%	Total	*p* (Chi^2^ test)
A. Crescents percentage groups and IS.						
Number	119	44	37	22	222	
Immunosuppressive treatment (data = 222)	36 (30.3%)	31 (70.5%)	29 (78.4%)	17 (77.3%)	113(50.9%)	0.000
Prednisone (data = 210)	36 (32.1%)	30 (69.8%)	27 (75.0%)	16 (84.2%)	109 (51.9%)	0.000
Methylprednisolone (bolus iv)	6 (6.2%)	11 (30.6%)	15 (42.9%)	10 (52.6%)	42 (22.5%)	0.000
Cyclophosphamide (data = 196)	2 (1.9%)	6 (14.6%)	7 (21.9%)	9 (50%)	24 (12.2%)	0.000
Azathioprine (data = 202)	17 (15.6%)	19 (47.5%)	21 (61.8%)	12 (63.2%)	69 (34.2%)	0.000
MMF (data = 206)	5 (4.6%)	1 (2.4%)	1 (2.7%)	2 (10.5%)	9 (4.4%)	0.665
B. Bivariate Cox regression analysis Risk to ESKD/KRT or death, in each group, according to IS (Reference No IS).						
HR	0.977	0.179	1.614	1.082		
CI 95%	0.379–2.521	0.033–0.980	0.344–7.587	0.300–3.903		
*p* Value	0.962	0.047	0.544	0.905		

ESKD/KRT: end-stage kidney disease/kidney replacement therapy; MMF: Mofetil mycophenolate; HR: Hazard ratio; CI: confidence interval

Coppo et al. [5] observed that those patients included in the VALIGA study who had received IS treatment showed a reduced predictive value of histologic lesions, although it usually persisted. In the IS group in this cohort, proteinuria and endocapillary hypercellularity were not associated with a higher risk of ESKD/KRT or death (as they were in the NoIS group) (Table 2A), possibly as a consequence of the IS treatment received, as other authors had observed [5,32]

Otherwise, tubule interstitial damage ≥25% was associated in all groups with the combined event in the multivariate risk analysis, likely because it was representative of chronic damage not reversible with IS therapy [32].

### Immunosuppressive treatment

As IS treatment remains controversial [10,17,18], the outcome was evaluated separately in the groups treated with IS and NoIS. The recently published TESTING study observed a better outcome in the group treated with low-dose methylprednisolone [49] if no remission was attained after standard supportive treatment. But serious adverse events had been observed with higher doses [49]. In this study, the IS group included all patients who received steroids with or without other immunosuppressants. Only 28.7% exclusively received steroids, as previously described, according to national guidelines [19], so all ISs were combined, despite the potential bias, as other retrospective cohort studies did [5,9,32].

The IS group did not show a significantly different rate of survival to the combined event (ESKD/KRT or death) (Figure 2), as STOP-IGA observed in a randomized clinical trial [15]. However, the IS group had more frequent baseline risk factors, such as higher proteinuria, glomerulosclerosis, and crescent percentages (Table 1).

National guidelines [19] suggest IS treatment when cellular or fibrocellular crescents are observed, even in less than 50% of glomeruli, similar to other authors [48,49] but slightly different from the recent KDIGO guidelines [10]. Beck et al. [50] reported that cyclophosphamide iv plus steroids in a group with inflammatory histologic damage (endocapillary and extracapillary hypercellularity) was associated with better outcomes (less proteinuria and higher GFR) than a standard treated group. Liang et al. administered methylprednisolone plus prednisone for 6 months to IgA patients with crescents <50%, and they observed proteinuria reduction [47] and concluded that IS treatment according to crescent presence was beneficial, as other authors did [37,38]. Crescents may improve with IS treatment [5,51], and mesangial and endocapillary hypercellularity and mononuclear interstitial infiltration [5,52] may also improve, as was reported in studies with a second KB. These treatments may interfere with the interpretation of retrospective studies that included treated patients (with unknown drug doses and durations), as was stated by Coppo [5] and Moriyama [32], and it likely occurred in this study. A significant association was also observed between higher crescent percentages and intravenous methylprednisolone bolus treatment (Table 3), and in the bivariate Cox analysis, in the group with 1–10% crescents, IS treatment was associated with better outcomes *vs.* the NoIS group (Table 3B), similar to the report by Haas and Trimarchi [17,18]. Additionally, in the IS group, neither proteinuria nor endocapillary hypercellularity was associated with a higher risk of ESKD/KRT or death, although they were associated in the entire population and in the NoIS group.

In the multivariate Cox regression analysis, in the NoIS group, the crescent percentage was not associated with a higher risk of the combined event. This may be explained by the fact that there were only five cases with crescent percentages ≥30% in this group because of the aforementioned national selection bias that considers crescents as an indication for IS treatment.

Most likely as a consequence of treatment, this cohort’s IS group achieved a similar outcome despite different, more severe, clinical and histological presentations. Therefore, it could not be excluded as a beneficial effect of IS therapy.

### Study strengths and limitations

The study has several limitations, as it was a retrospective analysis of a cohort with incomplete baseline clinical and histologic data, and neither time-average proteinuria nor eGFR slopes were available for analysis. As Oxford’s classification was published in 2004, most histologic descriptions did not include the precise mesangial proliferation magnitude or detailed glomerular sclerosis, so these data were not available. Treatment data were limited to the pharmacologic groups but did not include the exact dose, time of initiation, or duration of treatment in all cases. As most patients treated with IS drugs had received simultaneous steroids and other drugs, the efficacy of steroids alone could not be properly evaluated. Therefore, whether IS treatments were necessary and worth the risk could not be answered by this retrospective study, ongoing prospective clinical trials will be helpful.

However, the study also has strengths, as it was a study on a national cohort diagnosed by only two nephropathologists’ teams, looked after by nephrologists who applied national guidelines, and registered data on a mandatory registry (URG) maintained by a national prevention program (PPTG) with an extended follow-up of a minimum of 11 years. Additionally, hard outcomes such as ESKD/KRT and death were obtained from national mandatory registries. To the best of our knowledge, this is the oldest Latin American IgA cohort that reported a long follow-up outcome.

The national registry would allow future evaluation of the effect of new treatments that, hopefully, may improve IgAN prognosis.

## Conclusions

In a large Latin American IgAN cohort with an extended follow-up, the frequency and clinical and histologic presentations of IgAN were similar to those internationally reported, except that crescents were more frequent.

Renal survival at 20 years was 74.6%, which was not significantly different between those who were treated with immunosuppressors and those who were not.

The IS group (48.5%) had more severe clinical and histological baseline data.

Proteinuria diminished significantly in both groups.

The main independent risk factors for the combined event (ESKD/KRT or death) in the entire population and in the NoIS group were severe baseline proteinuria and tubule interstitial damage. In the IS group, tubule interstitial damage and ≥30% crescent percentages were independently associated with a higher risk.

The IS treatment administered to almost half the population, which included patients with more severe risk factors, obtained a similar outcome to the less damaged NoIS group.

Geolocation information gps coordinates of 34° 54′ 4.0032'' S and 56° 9′ 52.3152'' W.

## Supplementary Material

Supplemental Material

## Data Availability

Upon request, Dataset could be available as a Supplemental online archive.
