# Supplementary material for: Long-term follow-up of an IgA nephropathy cohort: outcomes and risk factors
Source: Ren Fail. 2023 Jan 23;45(1):2152694. doi: 10.1080/0886022X.2022.2152694 (PMC9873278; doi:10.1080/0886022X.2022.2152694)
Supplement: Supplemental Material [file IRNF_A_2152694_SM1509.pdf]

Supplemental Table 1. Survival to ESKD/KRT or death. **Crescents percentage 1-10% group (n=44).** Cox bi and multivariate regression analysis (adjusted to Endocapillary hypercellularity and immunosuppressive treatment).

	**Cox bivariate analysis**			**Cox multivariate analysis**		
	**HR**	**CI 95%**	**p**	**HR**	**CI 95%**	**p**
Age (years) [Continuous]	1.020	0.949-1.097	0.590			
Sex [Ref. male]	1.389	0.254-7.587	0.705			
BP initial [Ref < 130/80 mmHg,]	2.755	0.322-23.601	0.355			
eGFR initial [Ref > 60 ml/min/1,73m^2]			0.207			
30 - 60 ml/min/1,73m^2	1.734	0.194-15.525	0.623			
< 30 ml/min/1,73m^2	7.309	0.808-66.106	0.077			
Proteinuria initial groups [Ref. Mild]			0.505			
Moderate	0.329	0.034-3.168	0.336			
Severe	1.348	0.225-8.072	0.744			
Endocapillary hypercellularity [Ref. NO]	8.881	1.037-76.073	0.046	8.033	0.935-69.011	0.058
Tubule-interstitial damage groups [Ref.< 25%] 25-50%	3.539	0.647-19.369	0.145			
> 50%	No cases	--	--			
Immunosuppressive treatment [Ref. NO]	0.179	0033-0.980	0.047	0.202	0.037-1.108	0.066

ESKD=End stage kidney disease/KRT= Kidney replacement treatment, BP= Blood pressure, eGFR= estimated glomerular filtration rate, HR=Hazard ratio, CI= Confidence interval, Ref= Reference

Information Classification: General
